# Nanomedicine in Cancer Targeting and Therapy

**DOI:** 10.3390/jpm12081312

**Published:** 2022-08-13

**Authors:** Ilaria Elena Palamà, Stefano Leporatti

**Affiliations:** CNR-NANOTEC, Istituto di Nanotecnologia, Via Monteroni, 73100 Lecce, Italy

Currently, cancer represents a major cause of death in the world, despite all the progress made in developing new therapies. Gold standard therapies require the use of chemotherapeutic drugs associated with radiotherapy and the surgical excision of the localized tumors.

Unfortunately, chemotherapy/radiotherapy are not cell-specific but also affect healthy tissues, causing undesirable side effects. Furthermore, chemoresistance determines a reduction of clinical drug efficacy. Innovative therapeutic strategies are still required to overcome the intrinsic insensitivity of cancer cells. In this context, engineered nanostructured materials to specific target and kill cancer cells with a low drug dose, reducing the pharmacologic impact on healthy cells and their clonogenicity can pave the way towards new theragnostic strategies for cancer applications [1].

To this aim, recently it has been shown that the uses of different nanostructured materials for theragnostic applications [2,3,4] provide more functionality, representing a method of achieving a combined effect for cancer care [5]. Multifunctional nanostructured materials have the capability to carry out different active therapeutic molecules, maximizing drug efficacy with a single treatment, delivering therapeutic molecules to a specific place of action, and minimizing negative side effects [6,7,8,9,10,11]. In addition, the conjugation of nanostructured materials with targeting motifs/antibodies or imaging elements can be combined into a single nanostructure, enhancing the properties of materials with recognition capability and imaging [12]. 

In the last few years, researchers have focused on the activation of the immune system against cancer cells. Different nanostructured materials have been developed for this aim [13]. For example, nanoparticles have been developed to target the specific pathways of innate immune systems, such as Toll-like receptors, the programmed cell death protein 1, or the cytotoxic T lymphocyte antigen-4 [13,14]. 

Until now, existing clinical cancer therapy has not been applicable to all patients, principally due to the inadequate responses of individual patients caused by chemoresistance and/or immunosuppression. 

With this in mind, we are confident that this Special Issue will be able to explore ground-breaking approaches to nano-theragnostic therapy in cancer applications. In particular, we are expecting several contributions from the scientific community including mini-reviews, reviews, or research articles, which will focus on:

– the new synthesis of multifunctional nanostructured materials;
– the application of theragnostic materials in cancer treatment;
– the immune system and nanostructured materials.

Cancer nanomedicine is thus a promising novel area with significant future improvement potential, allowing physicians to use new nanoweapons in the universal war against cancer. Bearing this challenge in our mind, we are truly confident of stimulating new directions in the personalized treatment of cancer patients. 

## Data Availability

Not applicable.
